# Paralysis and delayed Z-disc formation in the *Xenopus tropicalis unc45b *mutant *dicky ticker*

**DOI:** 10.1186/1471-213X-10-75

**Published:** 2010-07-16

**Authors:** Timothy J Geach, Lyle B Zimmerman

**Affiliations:** 1 Division of Developmental Biology, MRC-National Institute for Medical Research, The Ridgeway, Mill Hill, London, NW7 1AA, UK

## Abstract

**Background:**

The protein components of mature skeletal muscle have largely been characterized, but the mechanics and sequence of their assembly during normal development remain an active field of study. Chaperone proteins specific to sarcomeric myosins have been shown to be necessary in zebrafish and invertebrates for proper muscle assembly and function.

**Results:**

The *Xenopus tropicalis *mutation *dicky ticker *results in disrupted skeletal muscle myofibrillogenesis, paralysis, and lack of heartbeat, and maps to a missense mutation in the muscle-specific chaperone *unc45b*. *Unc45b *is known to be required for folding the head domains of myosin heavy chains, and mutant embryos fail to incorporate muscle myosin into sarcomeres. Mutants also show delayed polymerization of α-actinin-rich Z-bodies into the Z-disks that flank the myosin-containing A-band.

**Conclusions:**

The *dicky ticker *phenotype confirms that a requirement for myosin-specific chaperones is conserved in tetrapod sarcomerogenesis, and also suggests a novel role for myosin chaperone function in Z-body maturation.

## Background

The sarcomere is the fundamental unit of the muscle myofibril, and mediates ATP-dependent contraction and relaxation. While the components of sarcomeres are well understood, the mechanics and sequence of their assembly remain under debate. A key early process in myofibrillogenesis is the polymerization of α-actinin-rich Z-bodies into the Z-discs that flank the myosin-containing A-band. In a mature vertebrate sarcomere, Z-discs are held in register with the central M-band by titin filaments tightly associated with filamentous actin [1,2].

Molecular chaperones, which mediate specific protein maturation events, have recently been shown to play important roles in vertebrate myofibrillogenesis. Originally identified as an **unc**oordinated nematode mutation [3,4], the muscle-specific chaperone unc45b cooperates with hsp90 to fold the N-terminal globular motor (S1) domain of sarcomeric myosins [5-7]. Depletion of these gene functions in zebrafish results in absence of integrated myosin in the sarcomere [8-11], but no effects on Z-disc formation were noted. While *in vitro *studies of these chaperones in cultured mammalian myoblasts have supported roles in sarcomere formation [6,12], *in vivo *requirements for these proteins have not been described in vertebrates other than teleost fish.

Vertebrate myofibrillogenesis is readily studied in *Xenopus *embryos, where most skeletal muscle arises from blocks of mesodermally derived somites running along the flank that eventually contribute to the easily-imaged transparent tadpole tail. Somites form and differentiate in a strict anterior-to-posterior sequence, permitting observation of different stages of muscle formation in a single embryo. Recently, a number of *Xenopus tropicalis *mutations displaying a range of developmental phenotypes [13-15] including defective cardiac muscle formation [16] have been described.

In one of these mutations, *dicky ticker *(*dit*^*mh71*^), embryos are completely paralyzed with no heartbeat. Tail muscle shows decreased birefringence under polarized light, consistent with disruption of sarcomeric structures. While the myofibrillogenesis program initiates and muscle myosin is detectable at early stages, sarcomeric myosin heavy chain (MyHC) staining rapidly decreases. Disorganized sarcomeres bounded by α-actinin-rich structures eventually form, but the transition from Z-bodies to Z-discs is delayed. In the first use of the rapid gynogenesis-based positional cloning strategy developed by Khokha et al, [17] we show here that the *dit *phenotype is caused by a missense mutation in the myosin chaperone *unc45b*. These results not only demonstrate a conserved role for this protein in vertebrate myofibrillogenesis, but also suggest a novel function for this chaperone in Z-disc formation, a key early step in sarcomerogenesis.

## Results

### A Cys to Arg conversion in the UCS domain of unc45b disrupts sarcomerogenesis in *dit*

*Dit *was first identified in a forward genetic screen for *Xenopus tropicalis *developmental mutations [13]. Homozygous embryos lack a heartbeat (Additional File 1, Movie S1) and are completely paralysed even after vigorous stimulation (Additional File 2, Movie S2), developing cardiac edema and dying by ~stage 45. When visualised under polarized light, *dit *somites showed a marked reduction in birefringence compared to wild type, suggesting disruption to the normal paracrystalline sarcomere structure (Additional File 3, Figure S1) consistent with defective muscle formation rather than aberrant innervation or metabolism. Phalloidin staining of stage 42 tail muscle likewise showed severe disruption to myofibrils (Additional File 3, Figure S1).

**Figure 1:**
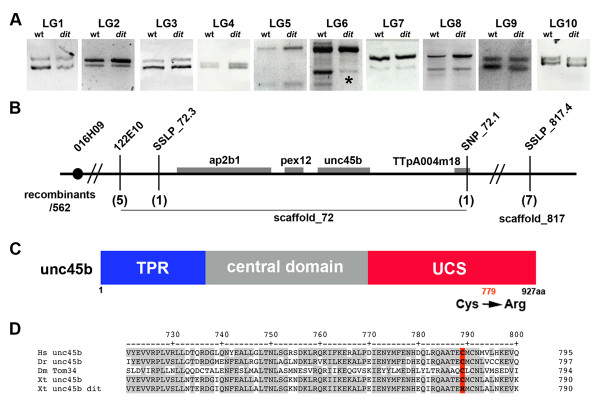
***dicky ticker *maps to a lesion in the myosin co-chaperone *unc45b***. **(A) **Pools of gynogenetically derived wild type and *dit *embryos were assayed for linkage to polymorphisms near the 10 *X. tropicalis *chromosomes. Clear linkage (*) was observed at Linkage Group 6 (Chromosome 2) **(B) **Linkage analysis using SSLP and SNP markers with 562 mutant embryos placed the *dit *locus in a 230 kb interval of Scaffold_72 containing four gene models including *unc45b*. **(C) **cDNA sequence of *dit unc45b *revealed a thymidine to cytidine transition, substituting Arg for Cys^779 ^in the UCS domain. **(D) **Cys^779 ^is phylogenetically conserved in *unc45b *orthologs from *Drosophila *(Dm) to human (Hs).

**Figure 2:**
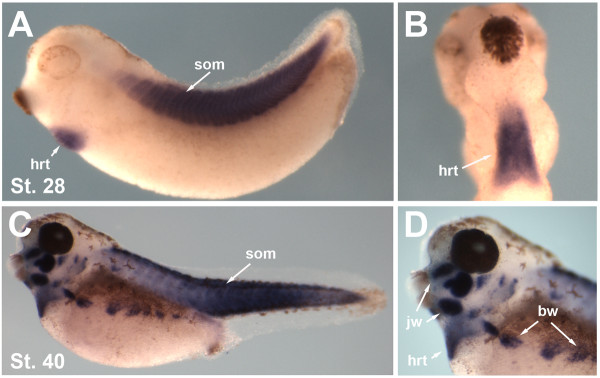
***Unc45b *expression in *Xenopus tropicalis***. **(A) **WISH showing *unc45b *expression in developing heart (hrt) and somites (som) at stage 28. **(B) **ventral view of heart expression at stage 28. **(C) ***unc45b *expression at stage 40 throughout the somites and **(D) **in jaw (jw), heart (hrt) and body wall muscles (bw).

The rapid gynogenetic mapping strategy described in Khokha et al. [17] was used to place *dit *onto a chromosome and estimate a locus-centromere distance. Briefly, *dit *and sibling wild type gynogenetic embryos were generated by 'early coldshock' suppression of polar body formation in haploid embryos derived from heterozygous carrier females. Bulk segregant pools of mutant and wild type gynogenotes were then assayed with centromeric SSLPs from the 10 *X. tropicalis *chromosomes [17]. Linkage was detected to marker 016H09 near the centromere of Chromosome 2/Linkage Group 6, but not to other centromeres (Figure 1A). An estimate of the mutation-centromere distance was obtained from the fraction of phenotypically mutant embryos observed (35%) in gynogenetic clutches, placing the locus ~15 cM from the centromere using the formula assuming complete interference (cM) = 50(1 - (2 × fraction mutant gynogenotes)) [18]. SSLPs from this region of the meiotic map as well as additional bespoke SSLPs and SNPs from identified scaffolds were then used for high resolution mapping on embryos obtained from a conventional cross of polymorphic heterozygous carriers. Analysis of 562 mutant embryos (1124 meioses) placed the mutant locus in a 230 kb interval on scaffold_72 (scaffold_72:967475-1197607) (Figure 1B) containing 4 gene models: *ap2b1*, *pex12*, *aldh3a1*, and the sarcomeric myosin chaperone *unc45b*, whose loss-of-function phenotype in zebrafish closely resembles *dit *[9]. Sequence of *unc45b *from wild type and *dit *embryos revealed a thymidine to cytidine transition at bp 2335 in the coding sequence, converting Cys^779 ^to Arg (Figure 1C). Conserved in both vertebrate and invertebrate unc45b orthologs (Figure 1D), Cys^779 ^is in the N-terminal UCS (**U**nc45 **C**ro1p **S**he4p) domain important for folding myosin *in vitro *[5,6]. Whole mount *in situ *hybridization (WISH) of wild type embryos shows *unc45b *is expressed throughout the somites and heart at stage 28 (Figure 2A &2B), becoming further localised to the jaw, branchial arches and body wall muscles by stage 40 (Figure 2C &2D). The mutation in a conserved cysteine residue, expression during muscle development, and similarity of loss-of-function phenotypes in zebrafish combine to suggest *unc45b *may underlie the *dit *phenotype.

### Z-disc formation is delayed in *dit*

**Figure 3:**
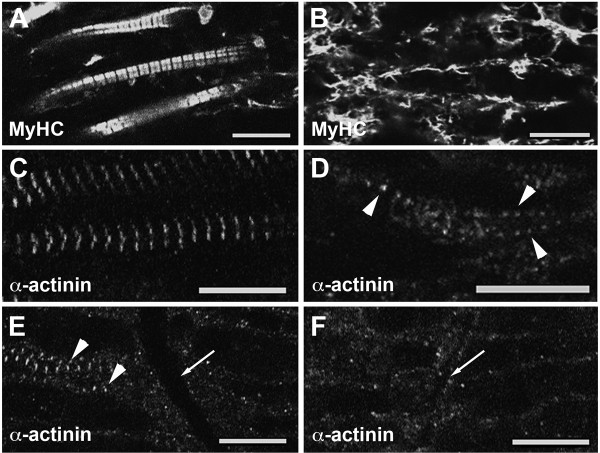
**Myofibrillogenesis is disrupted and sarcomere formation is delayed in *dit***. Confocal analysis of wild type (left panels) and *dit *embryos (right panels) for sarcomeric myosin heavy chain (MyHC) and Z-disc (α-actinin) immunoreactivity. **(A, C) **Anterior somites of stage 28 wild type embryos show sarcomeric myosin (MyHC) organized into A-bands separated by regularly spaced Z-discs (α-actinin). **(B) **Anterior *dit *somites show disorganized MyHC staining, and **(D) **α-actinin staining is punctate, with occasional ordered Z-bodies consistent with the appearance of incipient sarcomeres (arrowheads). **(E) **More posteriorly at st. 28, α-actinin staining shows wild type somites 15-16 (arrow indicates intersomitic boundary) are beginning to organize Z-discs (arrowheads), while **(F) ***dit *somites at this level show only background staining (arrow indicates intersomitic boundary). Scale bars 10 μm.

Myofibrillogenesis in *dit *was investigated further by confocal microscopy. At stage 28, the earliest at which *dit *can be identified, wild type and mutant embryos were scored, genotyped, and their sarcomeric structures analysed by immunohistochemistry. The oldest (most anterior) somites of wild type sarcomeres showed MyHC (detected by MHC A4.1025) integrating into evenly spaced A-bands (Figure 3A). *Dit *embryos at this early stage displayed MyHC staining in disorderly fibrillar structures at levels comparable to wild type siblings (Figure 3B), in contrast with the negligible MyHC immunoreactivity observed in zebrafish *unc45b *and *hsp90a *mutants [9,10]. Headless myosin species retain the capacity to form thick filaments [19], and it is known that *unc45b *functions to prevent myosin aggregation [20]. The MyHC staining seen in *dit *during early myofibrillogenesis may represent transient aggregates of filament intermediates in which head domains remain unfolded and are unable to integrate into the sarcomere.

We then evaluated Z-disc formation in *dit *embryos; Z-disc defects were not described in the zebrafish chaperone mutants *steif *and *sloth *[9,10]. In stage 28 *X. tropicalis *tadpoles, wild type anterior sarcomeres had formed discrete α-actinin-stained Z-disc structures (Figure 3C), while mutants lacked Z-discs but showed punctate staining consistent with α-actinin-rich Z-body precursors (Figure 3D). Occasional stretches of regularly spaced staining are seen consistent with the onset of sarcomere formation (arrowhead) similar to the premyofibrils described by Sanger et al. [21]. In more posterior somites, sarcomere formation is at an earlier stage. Figure 3E &2F show the border (arrows) between somites 15 and 16 in tails stained with α-actinin. In wild type (Figure 3E), Z-discs are beginning to assemble into sarcomeres in the slightly older somite 15 (arrowhead, left) while in the adjacent somite 16 no Z-discs are seen. No organized α-actinin staining is seen in mutant embryos at these stages in somite 15-16 (Figure 3F).

### MyHC is lost and Z-disc formation recovers in *dit *tadpoles

**Figure 4:**
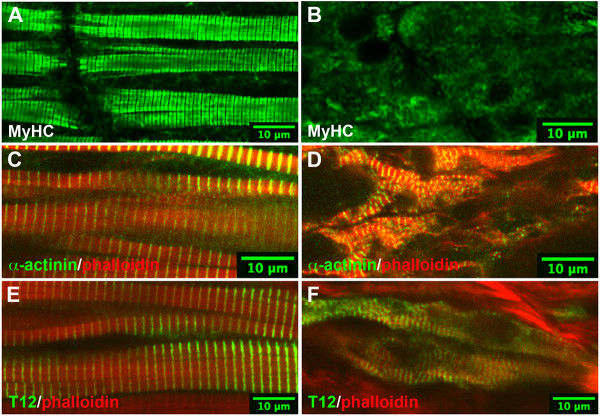
**MyHC immunoreactivity is lost and Z-disc formation recovers in *dit***. Confocal analysis of wild type (left panels) and *dit *embryos (right panels) stained with MyHC A4.1025, α-actinin and titin T12 antibodies. **(A, C, E) **A-bands and Z-discs are well-organized in wild type somites at stage 43. **(B) **Sarcomeric myosin staining in *dit *is virtually absent at these stages, but Z-disc-containing sarcomeres are observed **(D, F)**. Phalloidin stained thin filaments are present but disorganized in *dit *somites.

Later in development (stage 43), wild type sarcomeres are organized into paracrystalline structures with MyHC-stained A-bands, α-actinin-stained Z-discs, and titin-positive I-bands in register (Figure 4A, C, E). *Dit *tadpole tails at this stage showed no organized MyHC fibrils (Figure 4B), and the level of immunoreactivity was significantly weaker than wild type, more closely resembling the zebrafish myosin chaperone mutations [9,10]. Lower MyHC protein levels in *dit *were confirmed by western blots of stage 40 and 43 embryos using another pan-sarcomeric MyHC antibody, F59 (Additional File 4, Figure S2). Sarcomeric MyHC proteins are subject to ubiquitin-mediated degradation and autophagy [22-24], and it is likely that the misfolded head domains in *dit *are actively degraded. Interestingly, Z-discs (marked by both α-actinin and titin T12 antibodies) were present in *dit *tadpole tails at these stages, although these were scattered and severely out of register (Figure 4D &4F).

### Depletion of *unc45b *phenocopies *dit*

**Figure 5:**
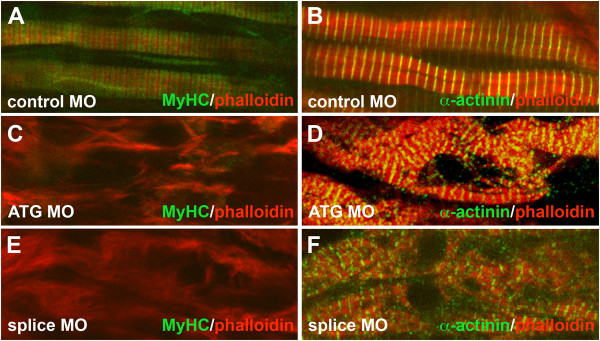
**Morpholinos against *unc45b *phenocopy *dit***. *Unc45b *knockdown closely resembles the *dit *phenotype and results in depletion of MyHC staining and disorganized sarcomeres. Confocal analysis of control MO **(A, C)**, ATG MO-injected **(B, D)**, and splice MO-injected embryos (**E, F) **for sarcomeric myosin heavy chain (MyHC) and Z-discs (α-actinin), co-stained with phalloidin.

To confirm that a deficit in *unc45b *function underpinned the *dit *phenotype, we injected two non-overlapping antisense morpholino oligonucleotides (AMO) into wild type embryos. Both a translation blocking (ATG-MO) and a splice blocking AMO deleting ~2/3 of the mature protein (Splice-MO) resulted in highly significant numbers of paralysed or partially paralysed tadpoles lacking heartbeat (ATG-MO 42/49 (p < 0.01); Splice-MO 32/34 (P < 0.01), compared to 0/52 in control AMO-injected embryos). Confocal analysis revealed that both the ATG-MO and Splice-MO produced specific defects in sarcomere formation mirroring those in *dit *(Figure 5), with reductions in sarcomeric MyHC staining and scattered α-actinin-rich Z-disc-like structures. The similarity of the mutant phenotype to those produced by these two independent knockdown experiments is consistent with *dit *being a strong hypomorph of *unc45b*.

**Figure 6:**
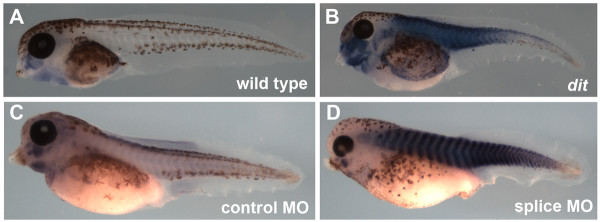
***unc45b *expression is upregulated in *dit***. WISH showing expression of *unc45b *at stage 43 in **(A) **wild type and **(B) ***dit*, and at stage 40 in **(C) **control MO and **(D) ***unc45b *splice-MO injected embryos. Both splice-MO injected and *dit *embryos show increased *unc45b *expression relative to controls.

Interestingly, WISH analysis shows that *dit *embryos and *unc45b *antisense-MO injected embryos express increased levels of *unc45b *mRNA relative to siblings or control MO-injected controls developed in parallel (Figure 6). In zebrafish *steif *(*unc45b*) and *sloth *(*hsp90a*) mutants, expression of the mutated genes is also upregulated [9,10]. The chaperone hsp90 regulates its own expression by holding the transcription factor hsf1 in a latent complex, which is released during heat shock or pharmacological stress when unfolded proteins compete for hsp90 binding [25-27]. The freed hsf1 in turn directly upregulates hsp90 as well as other stress response chaperones [28]. In the *dit *and *steif *mutants, persisting unfolded myosin head domains could likewise bind hsp90 and free a heat shock factor to increase chaperone transcription.

## Discussion

While the organization of the mature sarcomere is well understood, the order and mechanism of its assembly remain under debate [1,29]. Our data strongly suggest that Z-disc formation, a key early step in myofibrillogenesis, initially requires the presence of mature sarcomeric MyHC proteins. This observation most closely supports the premyofibril model proposed by Du et al. [30] in which closely spaced α-actinin-rich Z-bodies are initially separated by non-muscle myosin II; non-muscle myosin II is then replaced by sarcomeric MyHC proteins as these premyofibrils mature, coincident with Z-body alignment and polymerization into Z-discs (reviewed by Sanger et al. [29]). While closely spaced α-actinin-stained Z-bodies are observed in *dit *sarcomeres, lack of mature sarcomeric MyHC replacement could be responsible for delay of polymerization of these into Z-discs, and Z-disc alignment remains poor. Our data provide less support for the model proposed by Holtzer et al. [31], which hypothesizes that titin 'stitches' together α-actinin rich I-Z-I bodies with already formed thick filaments to add sarcomere elements at the ends of Z-disc containing striated myofibrils. This model does not anticipate the presence of the regularly spaced premyofibril-like α-actinin-stained Z-body structures observed in early *dit *sarcomeres. The molecular mechanism by which sarcomeric MyHC might participate in Z-disc maturation remains to be determined. While a direct interaction seems unlikely, feedback to Z-bodies via correct MyHC interdigitation with thin filaments or even contractile activity are also plausible explanations for the requirement for correct MyHC head domain maturation. Our data also do not rule out the possibility that *unc45b *functions directly in Z-disc maturation on an unknown protein target; indeed, unc45b protein is known to localize to mature Z-discs in zebrafish [32]. Alternatively, incorrectly folded MyHC heads or aggregates could interfere with Z-disc formation in the mutant, with this block released following myosin degradation.

## Conclusions

The *X. tropicalis dicky ticker *mutant demonstrates a conserved role for *unc45b *and sarcomeric myosin-specific chaperone function in tetrapod myofibrillogenesis, extending previous phenotypic analysis in teleost fish. A possible novel role for sarcomeric MyHC integration in Z-disc maturation is suggested by the observed delay in aggregation of α-actinin staining. *Dicky ticker *is the second *X. tropicalis *mutation to be cloned, and the first to make use of the rapid gynogenesis-based mapping strategy. Analysis of myofibrillogenesis mutations in *X. tropicalis*, with its potential for combining genetics with embryological and biochemical tools, promises additional insights into function and regulation of vertebrate muscle assembly.

## Methods

### Genetic Mapping of *dit*

Following generation of *dit *embryos by gynogenesis [17] or conventional matings, linkage was analyzed using standard amplification conditions and SSLP markers from the *X. tropicalis *meiotic map http://tropmap.biology.uh.edu[33], together with bespoke SSLPs and SNPs identified in sequence scaffolds of interest. Genomic sequences and scaffolds refer to Joint Genome Institute *X. tropicalis *Assembly v4.1 http://genome.jgi-psf.org/Xentr4. Centromeric markers were as described by Khokha et al. [17] except **LG4-C **(001E03), **LG8-C **(029D12), **LG9-C **(011H04) and

**LG3-C **scaffold_163:1658760-1659057),

(F:TTCAGCGAACAGAAGACAACA; R: CAGATGGAGAAATAGTGTTTCACTT;

Bespoke markers for high-resolution mapping were

**SSLP_72.3 **scaffold_72:967475-967913

F: TTGCCATAAAAGCAGCAGTG; R: TCGCATGCTCAAAATCACTC

**SSLP_817.4 **scaffold_817:274963-275158

F: TGGCTAGCGGTACCATAAAA; R: ACTTGGTAGGGGTGGCTCTT

**SNP_72.1 **scaffold_72:1185180-1197607.

F: CATCACGCATAGTCATTATGTTCTT; R: ATTATGGGATTGCTGGGTGA

A SNP in one allele creates a HaeIII restriction site in the amplified product, which was digested with HaeIII for 2 hours and resolved on a 3% agarose gel.

*Unc45b *cDNA was amplified using Platinum Taq (Invitrogen, UK). Sequencing was performed by Cogenics (UK) and analysed using Lasergene 8. Full-length wild type cDNA sequence is available at [GenBank: HM053434].

### Antisense Morpholino Oligonucleotides

A translation-blocking AMO (ATG-MO 5'-CCTTTAGCTGCACTGGGTCTTCCAT-3') and a splice-blocking AMO spanning exon-intron boundary 6 (splice-MO 5'-AGCACACATTATTCTTACCCAGTAC-3') were obtained from GeneTools LLC (Oregon, USA). GeneTools standard control AMO was used for control injections. All were diluted in nuclease free H_2_O and 5 ng injected into both blastomeres at the two-cell stage.

### Whole mount *in situ *hybridization

WISH was performed according to Harland et al. [34]. A 181 bp antisense probe was made from a 1217 bp fragment of *unc45b *(1444 - 2661 bp) cloned into pCRII-TOPO, linearised with HincII and transcribed with Sp6.

### Immunohistochemistry

Embryos were staged according to Nieuwkoop & Faber (1967), then fixed in 4% paraformaldehyde. Antibodies used were as follows: MyHC (MHC) A4.1025 [35] and Titin T12 (kind gifts from Elisabeth Ehler (KCL), α-actinin (EA-53, Abcam UK), and Alexa^® ^fluorophore conjugated secondary antibodies (Invitrogen, UK). F-actin was stained with Alexa^® ^543 phalloidin (Invitrogen, UK). Western blots were performed with anti-MyHC F59 [36], obtained from Developmental Studies Hybridoma Bank, University of Iowa, Iowa City, USA, and β-actin (Sigma, UK) using an HRP conjugated secondary IgG (Santa Cruz, USA). St 28 embryos were dehydrated in methanol and cleared in 2:1 benzyl alcohol:benzyl benzoate for visualization.

## Abbreviations

AMO: antisense morpholino oligonucleotide; Arg: Arginine; Cys: Cysteine; MyHC: myosin heavy chain; SNP: Single Nucleotide Polymorphism; SSLP: Simple Sequence Length Polymorphism; WISH: whole mount *in situ *hybridization.

## Authors' contributions

TG & LZ conceived the project, designed the experiments and wrote the paper. TG performed the experiments. Both authors read and approved the final manuscript.

## Supplementary Material

Additional file 1 ***dit *embryos lack heartbeat**. Beating heart at stage 40 in wild type (top) is not seen in *dit *(bottom). Click here for file

Additional file 2 ***dit *tadpoles are paralyzed**. Wild type tadpoles at stage 40 are motile (top); *dit *tadpoles (bottom) are paralyzed. Click here for file

Additional file 3 **Muscle structure is disrupted in *dit *tails**. **(A) **Birefringence of polarized light in stage 43 wild type tail. **(B) **Birefringence is greatly reduced in *dit *tails of the same stage. **(C) **Phalloidin staining of stage 43 wild type tail muscle showing orderly myofibril structure. **(D) **Phalloidin staining in *dit *embryo tails shows disorganized myofibrils. Click here for file

Additional file 4 **Western blot analysis of wild type and *dit *embryos with MyHC F59 antibody**. Presence of MyHC as detected by the F59 antibody is reduced in *dit *compared to wild type embryos at both stages 40 and 43. Click here for file
