# Supplementary figures and images for: Paralysis and delayed Z-disc formation in the Xenopus tropicalis unc45b mutant dicky ticker
Source: BMC Dev Biol. 2010 Jul 16;10:75. doi: 10.1186/1471-213X-10-75 (PMC2919470; doi:10.1186/1471-213X-10-75)

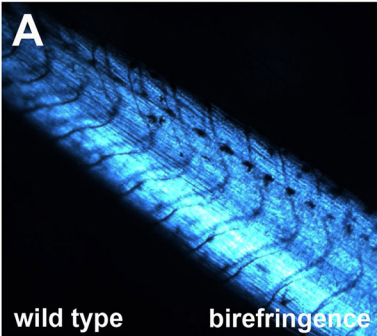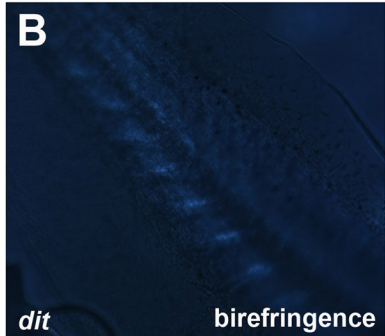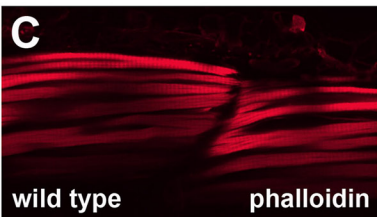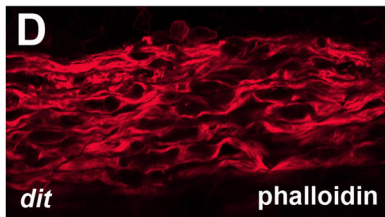

Supplement: Additional file 3 — Muscle structure is disrupted in dit tails. (A) Birefringence of polarized light in stage 43 wild type tail. (B) Birefringence is greatly reduced in dit tails of the same stage. (C) Phalloidin staining of stage 43 wild type tail muscle showing orderly myofibril structure. (D) Phalloidin staining in dit embryo tails shows disorganized myofibrils. [file 1471-213X-10-75-S3.PDF]

stage

wild type  
dit

40

wild type  
dit

43

F59

$\beta$ -actin

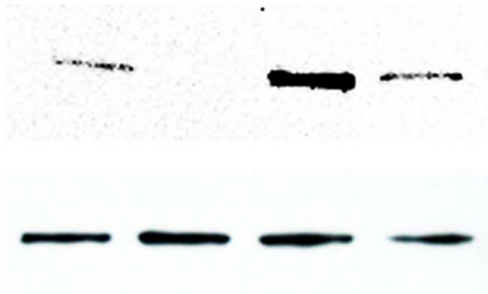

Supplement: Additional file 4 — Western blot analysis of wild type and dit embryos with MyHC F59 antibody. Presence of MyHC as detected by the F59 antibody is reduced in dit compared to wild type embryos at both stages 40 and 43. [file 1471-213X-10-75-S4.PDF]
